# Factors related to suicide’s unpredictability: a qualitative study of adults with lived experience of suicide attempts

**DOI:** 10.1080/17482631.2019.1650585

**Published:** 2019-08-10

**Authors:** Jacqueline K. Krychiw, Erin F. Ward-Ciesielski

**Affiliations:** Department of Psychology, Hofstra University, Hempstead, NY, USA

**Keywords:** Suicide, actor-observer bias, biosocial theory, interpersonal-psychological theory of suicide

## Abstract

**Purpose**: In recent decades, suicide prevention initiatives have increased substantially, yet the suicide rate has continued to rise, and suicide deaths are still generally perceived as unexpected. This study sought to identify factors that might account for this discrepancy by focusing on the exhibition of suicide warning signs.

**Methods**: Qualitative interviews were conducted with 15 adults [mean age = 36 (SD = 14), 93% female] who had attempted suicide at least once in their lifetime.

**Results**: A disconnect between participants and their environment emerged as a central theme. Many expressed ambivalence about whether they wanted others to intervene before their attempts, resulting in either expression or inhibition of warning signs. Regardless of whether they wanted their attempt to be predictable, most participants expressed disappointment if they perceived a lack of intervention before their attempt. In some cases, this disappointment exacerbated distress and may have contributed to the attempt itself. Participants also expressed difficulty disclosing their suicidal ideation to others. Thus, even if they wanted help, participants were unsure how to effectively attain it.

**Conclusions**: Findings underscore the complexity of predicting and preventing suicide; however, engaging individuals with lived experience in these efforts facilitates greater understanding toward outreach and intervention approaches.

Over the past twenty years, the rate of suicide fatalities has increased substantially; the age-adjusted suicide rate increased by 33% from 10.5 to 14.0 per 100,000 from 1999 through 2017. (Centers for Disease Control and Prevention [CDC], 2018). This increase, in addition to the general categorization of suicide as an unexpected form of dying (Bailley, Kral, & Dunham, 1999; Ellenbogen & Gratton, 2001), highlights a significant public health concern in need of extensive research attention. Over the past several years, global suicide prevention initiatives have increased substantially to try and address this dilemma (Arensman, 2017). The World Health Organization (WHO), the International Association for Suicide Prevention, and the American Association of Suicidology (AAS) are just a few of the organizations that have created action plans, a World Suicide Prevention day, and websites listing common warning signs (i.e., specific signs that have the potential to be noticed by others and suggest imminent intent to die) and risk factors to spread suicide awareness (AAS, 2017; Arensman, 2017; Dedić, 2016; Rudd et al., 2006). The “IS PATH WARM?” mnemonic is another example of an effort to ease public recognition and recollection of common warning signs (i.e., suicide **i**deation, **s**ubstance abuse, **p**urposelessness, **a**nxiety, **f**eeling **t**rapped, **h**opelessness, **w**ithdrawal, **a**nger, **r**ecklessness, and **m**ood changes; AAS, 2017). Additional studies have explored cognitive warning signs for suicide, including state hopelessness, conceptualizing suicide as a solution, fixation on suicide, focus on escape, and loneliness (e.g., Adler et al., 2016). However, despite these organizational efforts to raise awareness, the general public may not understand what to look for as “warning signs” of suicide (Latakiene, Skruibis, Dadasev, Grizas, Dapseviciute, & Gailiene, 2016). An analysis of suicide communication processes in a Lithuanian study supports this claim (Latakienė et al., 2016). Findings suggest that although suicide attempters tried to reach out to others prior to or after their attempt, only when the expression of warning signs was made *during* an actual attempt, did other people end up actively trying to prevent the attempt by going to the location of the act, calling an ambulance, or organizing help from a distance. In fact, the most common reactions to disclosure prior to an attempt were disbelief and general unresponsiveness. It seems that individuals surrounding the attempters were not aware of the seriousness of intent (Latakienė et al., 2016) and thus, may have overlooked important warning signs. Consequently, this lack of support was a compelling contributing factor to heightened risk of suicide.

Therefore, increasing public awareness of common warning signs through active didactics has been a focus of suicide prevention efforts, instead of relying on a passive list of potential signs. Madson and Vas (2003) conducted an activity-based task including fictional vignettes in a college psychology course to assess the participants’ abilities to point out risk factors associated with suicide. Importantly, after a discussion about the “correct” answers—which established which person was most likely to die by suicide—participants in the study demonstrated improved ability to recognize risk factors on a subsequent assessment. Several additional studies also indicate the potential effect of educational intervention on understanding signs of risk (Lamis, Underwood, & D’Amore, 2016; Pisano, Cross, Watts, & Conner, 2011; Tsai, Lin, Chang, Yu, & Chou, 2011). Although the public seems to benefit from learning about common warning signs in order to identify them more readily (e.g., Lamis et al., 2016; Madson & Vas, 2003; Pisani et al., 2011; Tsai et al., 2011), research suggests that the “IS PATH WARM?” model is not a valid way to predict who will attempt suicide (Lester, McSwain, & Gunn, 2011). Similarly, other publicly available lists of warning signs are not evidence-based and do not appear to be situated within a theoretical framework. This is largely a reflection of the limitations in our ability to predict suicide attempts and deaths more generally. For instance, a recent meta-analysis of 50 years of research regarding risk factors of suicide has indicated that present risk factors for suicidal thoughts and behaviors are weak and erroneous (Franklin et al., 2017). Despite substantial efforts, results also suggested that the predictive ability of recognizing individuals at risk of suicide has not improved over the past 50 years (Franklin et al., 2017).

Many warning signs require observing the suicidal individual and recognizing expressions or communications of potential risk (e.g., AAS, 2017); however, the rising suicide rates suggest this approach to recognizing risk may be insufficient. Although there is research suggesting that the majority of people who attempt suicide communicate directly and/or indirectly about their ideation and intent prior to their attempt (Hawton, Houston, & Shepperd, 1999; Robins, Gassner, Kayes, Wilkinson, & Murphy, 1959; Rudestam, 1971), suicide remains difficult to predict. Therefore, it is important to examine the discrepancy in reported rates of communication and the perception that suicide is unpredictable. At least three potential issues may be at play: 1) communication happens but is misinterpreted, unrecognized, and/or expressed in ambiguous ways, 2) communication happens but is invalidated (e.g., not taken seriously, lack of intervention), and 3) communication does not happen due to the anticipation of stigmatizing reactions and/or fears of placing burden on others. The complexity of circumstances prior to suicide attempts are considerable (e.g., attempts may even be surprising to the attempter; Ghio et al., 2011); however, several theories may help to explain the discrepancies between displays or perceptions of suicide warning signs and prospective prediction of suicidal behaviors. Specifically, the actor-observer bias, the biosocial theory, and the interpersonal-psychological theory of suicide provide potentially useful frameworks from which to view these multifaceted interpersonal dynamics.

Although it has been demonstrated that suicidal individuals may neither perceive a need for help nor want it (e.g., Bruffaerts et al., 2011; Czyz, Horwitz, Eisenberg, Kramer, & King, 2013), it is possible that some suicidal individuals are communicating about their risk, but signs are being misinterpreted or unrecognized. Specifically, perhaps the warning signs that attempters believe they are exhibiting are not as salient to others as they are to the attempters themselves. This may indicate that perception or observer expectation plays an important role in recognizing the warning signs of suicide. Jones and Nisbett (1972) posited that there are different ways in which individuals understand an event based on whether they are actively participating in the event, or simply observing the event. This actor-observer bias suggests that actors tend to attribute causes to situational factors (e.g., I fell because the road was icy), while observers tend to attribute causes to personal factors (e.g., she fell because she is clumsy). Although substantial research has been dedicated to exploring this effect, results are inconsistent (Aronson, 2002; Fiske & Taylor, 1991; Jones, 1976; Malle, 2006; Watson, 1982) and more research in different contexts is needed. For instance, applying this theory to the experience of a suicide attempter, it is possible that the suicide attempter (actor) might believe they are expressing warning signs and are yearning for their environment to aid them in feeling better, yet those in their environment (observers) may think the expression of possible warning signs are simply characteristics of their personality, not realizing that the suicide attempters may be trying to elicit help from those around them. As a result, it is possible that suicidal individuals believe they are directly communicating about their risk, yet might be expressing ambiguous signs (Robins et al., 1959); if this is the case, a discrepancy between the attempter and observers may result in missed opportunities for intervention.

Few studies to date have explored this concept within suicide-specific contexts and the few that have, are over three decades old, leaving several unanswered questions. One study investigated responses from hospital physicians regarding, among other things, circumstances under which they would kill themselves. Results demonstrated that the primary drive for participants’ hypothetical suicides was the presence of an incurable disease, supporting the importance of personal factors when “actors” are evaluating possible causes for events; factors associated with relationships (situational factors) were assigned a secondary causal role (Reimer & Doenges, 1981). Another study sought to explore differences in perceptions of why individuals might attempt suicide (Goggin, Range, & Brandt, 1986). Participants were randomly divided into two groups, each reading a one-paragraph vignette about a female that died by suicide and her sister. The two groups differed only in that one group was asked to imagine that they were the suicidal female in the story (actor role) and the other was told the story was about a female named Jeanne (observer role). The “actor” group attributed the cause of their suicide to be the psychological disturbance of their “sister” (situational factor). Although these results provide promising support for this actor-observer paradigm in relation to perceptions of suicide, in both studies, participants had no history of suicide attempts and were asked to complete self-report questionnaires/vignettes about *hypothetical* situations that included suicidal individuals and their environmental circumstances (Goggin et al., 1986; Reimer & Doenges, 1981). Participants also *imagined* themselves as suicide attempters to create the “actor” bias, rather than having personal lived experience. Importantly, a third study of the actor-observer bias *did* include suicide attempters (Hawton, Cole, O’Grady, & Osborn, 1982) and demonstrated promising results by comparing adolescent suicide attempters’ reported motivations for overdosing with the perceptions of their clinical assessors. Hawton et al. (1982) found that clinical assessors (observers) were more likely to perceive a suicide attempt as “manipulative” (personal attribution), while the suicide attempters (actors) reported the urge to rid themselves of unpleasant feelings and/or to demonstrate distress as a result of relationship stressors (situation attribution). Although these findings support the potential actor-observer bias in suicidal populations, additional research is needed to replicate these results, further research this potential dynamic in lived experience samples, and explore whether the actor-observer bias can explain why suicide is still generally seen as an unexpected cause of death (Bailley et al., 1999; Ellenbogen & Gratton, 2001) to loved ones left behind (observers). Specifically, warning signs exhibited by suicide attempters (actors) may be overlooked or misinterpreted as a result of differing perceptions of the same situation.

This possible inconsistency between the perception of those suffering—who think their suffering is clear and obvious to others—and the individuals around them—who do not notice the suffering or do not take it seriously—may result in the perception of an invalidating environment for suicidal individuals. It is also possible that individuals observing warning signs, recognize them and understand them to be serious, but for some reason (e.g., not knowing what to do) do not act on them (e.g., Wolk‐Wasserman, 1986). In either case, the way others respond to communication may affect the likelihood of communication occurring again in the future. The biosocial theory suggests that a combination of biological factors and a dysfunctional or invalidating environment can result in the development of psychopathology and dysfunctional behaviors (Linehan, 1993). Therefore, it is possible that invalidating responses to the expression of warning signs can increase urges to die by suicide. Consequently, suicidal individuals may intentionally begin to suppress or obscure the expression of warning signs because they believe they won’t be met with favorable reactions. One study, involving Turkish and Swedish adolescents, provides support for this hypothesis; results demonstrated that many suicidal individuals did not disclose their suicidal urges because they believed they couldn’t tell anyone in their life, believed no one could help them, and were fearful of judgmental reactions (Eskin, 2003). Veiel, Brill, Hafner, and Welz (1988) provided further evidence to support the claim that anticipated reactions from others might greatly impact whether or not suicidal individuals disclose their urges to die by suicide. Researchers found that although daily positive interactions were important for suicidal individuals to feel socially integrated, they were not the most important aspect of social interaction. Notably, crisis support was found to reduce the impact of stressful effects in suicidal populations. This suggests that perceived validation during a crisis (i.e., taking the crisis seriously, offering support, responding in a validating manner), rather than simply having an available social network, may play a particularly important role for suicidal individuals. The number of individuals who can provide this crisis support may be small and thus, potential attempters may only express warning signs to certain people, leaving others unaware of their circumstances.

Myriad research has also shown that negative attitudes and stigma toward suicide attempters are pervasive across community and clinical settings (Binnix, Rambo, Abrutyn, & Mueller, 2018; Lester & Walker, 2006; Pompili, Girardi, Ruberto, Kotzalidis, & Tatarelli, 2005). In order to avoid stigmatization, it is probable that suicidal individuals choose not to communicate warning signs. In fact, suicide attempt survivors experience self-stigma in addition to stigma from others (Sheehan, Corrigan, & Al-Khouja, 2016). In one study, an attempter believed he was “weak” and another thought “something was wrong with him” (Sheehan et al., 2016). Sheehan et al. (2016) also found non-fatal suicide attempters were commonly seen as attention-seeking, selfish, incompetent, emotionally weak, and immoral. Additionally, attempters in another study indicated feelings of stigma related to overgeneralization about the severity of their attempt, creating feelings of hopelessness regarding their recovery (Rimkeviciene, Hawgood, O’Gorman, & De Leo, 2015). It is apparent that feelings of stigma experienced by suicide attempters may even further increase risk of subsequent suicidal behaviors (Oexle et al., 2018).

In addition to the anticipation of unfavorable responses (e.g., invalidation, lack of intervention, stigma), feelings of burdensomeness may also contribute to suppression of warning signs by suicidal individuals. Joiner’s interpersonal-psychological theory of suicide (2005) posits that the presence of thwarted belongingness (the belief that one does not belong) and perceived burdensomeness (the belief that one’s existence creates a burden on loved ones) leads to the desire for suicide. In combination with the acquired capability to make the attempt, these experiences lead to an increased probability of attempting suicide. A recent comprehensive review of twenty-seven research studies involving clinical samples supports this theory, demonstrating consistent associations between perceived burdensomeness and both suicidal ideation and suicide attempts (Hill & Pettit, 2014). Taking this theory into account, it is possible that suicidal individuals, who often might already feel like they are a resource liability, struggle to communicate their suicidal thoughts and/or behaviors because they do not want to worry others or put pressure upon others to intervene. Thus, suicidal individuals may suppress signs that they are contemplating suicide to prevent further feelings of being a burden on others. Findings from a recent qualitative study exploring the motivation behind choosing to disclose suicidal urges has supported this hypothesis (Fulginiti & Frey, 2018). Specifically, results indicated that individuals with lived experience that reported increased tendencies to disclose their suicidal urges also reported lower levels of burdensomeness prior to disclosure (Fulginiti & Frey, 2018).

In summary, there are several possible explanations for the discrepancy between increased suicide prevention initiatives alongside the continued perception of suicide as unexpected to loved ones left behind. It is possible that attempters *do* exhibit warning signs; however, others don’t recognize, understand, and/or validate them, creating an invalidating environment. It is also possible that attempters gradually begin to suppress signs because of feared stigma from others and/or feelings of burdensomeness. This in turn may lead to increased difficulty for potential observers to recognize warning signs and suicide risk. It is also possible that attempters try to suppress their suffering from the start, resulting in loved ones not being given the opportunity to realize individuals are at imminent risk.

The WHO has estimated that more than 800,000 individuals die by suicide each year (Dedić, 2016); thus, past approaches to identify and understand the warning signs of suicide may be insufficient. Past research has indicated that the majority of individuals who attempt suicide communicate directly and/or indirectly about their ideation and intent prior to their attempt (Hawton et al., 1999; Robins et al., 1959; Rudestam, 1971), yet suicide remains difficult to predict. Therefore, investigating relevant factors associated with risk by speaking directly to suicide attempters may be most enlightening. These individuals are best suited to provide information about their exhibition of warning signs prior to an attempt and may provide insight as to why deaths by suicide are still seen as predominantly unexpected. Speaking directly to suicide attempters also provides an important opportunity to understand the “actor’s” point of view which enables us to evaluate the presence of the actor-observer bias without having to use hypothetical vignettes and participants’ imaginations. Guided by the prior literature, the aim of the current study was to understand what factors contribute to the discrepancy between known warning signs and suicide predictability using qualitative interviews with adults with lived experience of suicide attempts. Conducting qualitative research is vital within this population (Cutcliffe, 2003) and allows attempters to discuss their unique perceptions of the warning signs that they may or may not have exhibited to others, what led to their decisions to disclose or withhold, and the reactions they received.

## Method

### Participants

Participants included 15 adults [mean age = 36 (SD = 14), 93% female] living in the USA who had made at least one lifetime suicide attempt; occupations were varied (e.g., student, retired, mental health worker, cashier, professor). Seven out of 15 participants reported high lethality attempts (i.e., medical intervention needed to treat the effects of the attempt). Participants’ demographic information is provided in Table I. Participants were recruited via the American Association of Suicidology (AAS) listserv and social media forums (e.g., Twitter, Facebook, & LinkedIn) through advertisements introducing the purpose, inclusion and exclusion criteria, and compensation information for the study. To abide by ethical standards, potential participants were made aware that this investigation was for those who had lived experience (i.e., individuals who have attempted suicide at least once in their lifetime), entailed a phone interview that would be recorded, and would not involve psychotherapy. Inclusion criteria included living in the USA, being 18 years or older, having made at least one suicide attempt, and being willing to be audio-recorded. Exclusion criteria included suicidal ideation in the past month (defined as endorsing any item on the Columbia Suicide Severity Rating Scale Screener; Posner et al., 2008) and being unwilling to consent to study procedures and/or recording. The present study was approved by the institutional review board of a northeastern university in the USA.

**Table I.** Participant characteristics (N = 15).

	n	%
Gender		
Female	14	93.0
Male	1	7.0
Sexual orientation		
Heterosexual	9	60.0
Homosexual	1	7.0
Bisexual	2	13.0
Other	3	20.0
Ethnicity		
Caucasian	13	87.0
More than one race	2	13.0
Highest education		
High school or equivalent	2	13.0
Some college	6	40.0
Bachelors’ or Associates degree	3	20.0
Beyond bachelors’ degree	4	27.0
Annual household income		
Less than $25,000	3	20.0
$25,000–$50,000	6	40.0
$50,001–$75,000	2	13.0
More than $75,000	4	27.0
Number of lifetime suicide attempts		
1	2	13.0
2	1	7.0
More than 2	8	53.0
Not reported^a^	4	27.0
Lifetime NSSI (yes)	8	53.0
Not reported	4	27.0
Means used for first attempts		
Overdose/self-poisoning	14	93.0
Transportation	1	7.0
Participant psychological disorders		
Anxiety	6	40.0
Depression	6	40.0
Bipolar	3	20.0
Posttraumatic stress	4	27.0
Borderline personality	4	27.0
Other	3	20.0
Not reported	4	27.0
Family psychological disorders		
Bipolar	6	40.0
Depression	6	40.0
Anxiety	4	27.0
Substance use	3	20.0
Posttraumatic stress	2	13.0
Other	2	13.0

NSSI = non-suicidal self-injury.

^a^All participants were required to have made at least one suicide attempt; however, the number of lifetime suicide attempts for these participants is missing.

### Procedure

Interested potential participants were asked to respond to advertisements via email with their full name, phone number, and home address. This information was requested to ensure appropriate intervention was possible if an individual was deemed at imminent risk for suicide during the screening assessment. Although it was not specifically requested that participants complete the interview at their homes, only one participant reported being at a location other than their primary address during the interview and was willing to provide information regarding their whereabouts if risk was imminent; this participant was deemed safe and thus, gathering information regarding their whereabouts was not necessary. Email contact from potential participants prompted the researchers to provide additional information about the study and a consent form. If participants provided electronic informed consent, a phone interview was scheduled. Phone interviews took place within a private office setting, using a white noise machine to ensure confidentiality. Interviews consisted of a pre-interview stress assessment, demographic and eligibility questions, interview questions regarding participants’ suicide attempt(s), a post-interview stress assessment, and, if deemed necessary, a suicide risk assessment. Each interview lasted approximately 45–60 minutes. Procedures were in place for ineligible and distressed participants; however, no participants required use of these procedures.

All questions were focused on participants’ first suicide attempt to eliminate the possibility of a previous attempt as a confounding warning sign. To ensure clarity, participants were also given our definition of a suicide attempt (i.e., “intentionally causing harm or injury to yourself with the intent to die as a result of your actions”). The previously mentioned definition of “warning sign” was also provided (i.e., “specific signs that have the potential to be noticed by others and suggest imminent intent to die”). The interviewer typed notes throughout the call using a secure online survey (i.e., Qualtrics, www.qualtrics.com). All phone conversations included consent breaks to ensure that the participants understood their involvement was voluntary and they could withdraw from the study at any point. Participants were also treated with sensitivity throughout the call and given the opportunity to provide the interviewer with feedback about their experience during a debriefing procedure, regardless of whether participants spontaneously reported distress following completion of the phone interview. All individuals who took part in any portion of the interview were provided with crisis hotline phone numbers and national mental health services information.

### Measures

#### Stress assessment

The University of Washington Risk Assessment Protocol (UWRAP; Linehan, Comtois, & Ward-Ciesielski, 2012) was utilized to monitor participants’ stress throughout the phone interview. The pre-assessment sections gather information about the participant’s current levels of emotional distress, urges to engage in self-injury, urges to die by suicide, and urges to use drugs and alcohol on a Likert-type scale from 1 (low) to 7 (high); total scores range from four to 28. The pre-assessment procedures also include generating a plan with the participant for what to do if domain ratings increase or if distress arises during the interview. Following completion of the interview, the post-assessment procedures include reassessing each domain and comparing these to pre-assessment levels. If a participant reported increased or elevated self-injury or suicide urges, the interviewer would have completed a suicide risk assessment (if indicated) and/or employed the mood improvement protocol as necessary. This was not necessary for any participant. UWRAP-pre total mean score = 6.3 (SD = 3.0), range = 4–10 and UWRAP-post total mean score = 6.1 (SD = 1.6), range = 5–9 suggest that interviews were not substantially stressful for participants.

#### Demographics

Individuals provided information regarding their age, gender, ethnicity, sexual orientation, highest level of education, occupation, familial history of psychiatric disorders, and present financial status. Based on the definition stated previously, individuals were then asked about their lifetime history of suicide (e.g., “How many times did you attempt suicide”) and lifetime history of non-suicidal self-injury (e.g., “Have you ever engaged in non-suicidal self-injury, i.e., intentionally causing harm or injury to yourself with no intent to die as a result of your actions?”). Lastly, participants were asked to provide information regarding their lifetime history of psychiatric diagnoses (e.g., “Were you ever diagnosed with a psychiatric disorder?”).

#### Qualitative interview

Participants were asked a series of predetermined questions about their experiences prior to their first suicide attempt. Participants rated the clarity of their memory for their first attempt (from 0 to 5, all participants rated clarity at least 3 out of 5). Descriptive information about the impulsivity of the attempt, contributing factors that lead to the attempt, and the level of medical or psychiatric care needed after the attempt was obtained. In addition, open-ended inquiries about warning signs were discussed. These included whether or not attempters believed they exhibited warning signs, the reactions they received from others related to the exhibitions, and whether or not attempters wanted their attempts to be predictable to those around them. Specific questions to begin the discussion in each interview domain are provided in Table II.

**Table II.** Phone interview questions.

Domains	Specific questions asked
Clarity of memory	“Please rate from 1 (very poor) to 5 (very clear) how clear your memory is regarding your experiences prior to your first suicide attempt."
Impulsivity	“Did you plan your first suicide attempt (yes or no)?”
Contributing factors	“What were the immediate things that you believe contributed to this attempt?”
Level of medical intervention	“What level of medical intervention was required after this attempt?”
Exhibition of warning signs	“Do you feel you exhibited any warning signs before this attempt? Why or why not?”
Predictability of attempt	“Do you think your attempt was predictable?”
Intentions regarding predictability	“Did you want it to be predictable? Why or why not?”
Family history	“Please tell me any medical or psychological family history that you think may be relevant.”
Additional information	“Are there any other pieces of information related to the things we have talked about that you think I have missed?”
Debrief	“How was this experience for you?”

### Data analysis

Audio-recordings of each interview were transcribed. Interpretative phenomenological analysis (IPA; Smith, Jarman, & Osborn, 1999) was used. This procedure involves five steps that allow themes to emerge from data, focuses on what matters to participants, and subsequently investigates what these important things mean to participants. Before coding began, three coders reviewed IPA and familiarized themselves with the appropriate analytic guidelines. The first four phases of IPA require coders to analyze the data independently. These phases involved 1) a “free-read” through one of the transcripts, noting any biases and/or judgments that came up in response; 2) a thorough read of the same transcript and note of overall themes of the participant’s story; 3) an attempt to make sense of the emerging themes within one transcript, clustering them into relational frameworks to create superordinate (broad themes identified by participants) and subordinate (more narrow themes that fall within superordinate themes) themes; 4) an analysis of other cases, following steps 1 through 3 for each transcript. The fifth and final step involved coders meeting to integrate all major themes identified within each transcript and organizing them into the most prevalent themes.

## Results

The present study sought to gain a better understanding of why suicides are still generally categorized as unexpected forms of dying and predicting suicide fatalities is so complex. Using IPA, “a disconnect between suicidal individuals and their environment” was identified as the central theme related to participants exhibition of warning signs prior to their first suicide attempt. Several subordinate themes were also identified. Overall, ten participants reported suppressing warnings signs and five participants reported fully exhibiting warning signs prior to their first attempt. In total, only two participants reported wanting their attempts to be predictable to others and nine participants indicated that their first attempt was impulsive.

### A disconnect between suicidal individuals and their environment

Participants indicated that they suppressed and/or obscured the exhibition of warning signs because of unpredictable or unfavorable environmental factors. They reported ambivalence regarding whether or not they wanted help, disappointment if they perceived that others did not try to intervene, and difficulty communicating directly to others about their thoughts of suicide.

#### Ambivalence regarding help

Nine participants expressed ambivalence about whether they wanted help from individuals in their lives prior to their attempts. This resulted in a variety of different expressions of warning signs or lack thereof (e.g., wanting others to intervene/help, turning away help offered). Some participants suppressed potential warning signs from the start, fearing how others would react if they exhibited signs indicating they were in imminent danger and needed support. Others displayed warning signs to certain individuals in order to gauge reactions. Importantly, many ultimately masked signs that might have suggested they were suffering as a result of being disappointed by prior responses. Feelings of invalidation, beliefs that others wouldn’t take them seriously, and fears of being stigmatized were frequently mentioned. Additionally, participants often reported that invalidation exacerbated urges to die by suicide.

“I told my husband that I was suicidal, but he was very determined to leave the marriage. He just said like, ‘I don’t care.’ I don’t think I would have gone on to make further attempts or maybe not as many. I really didn’t get any support after that.”

“I was just so exhausted and tried to have a universal poker face and that would just make me more tired. I was trying to keep it up, mostly for the sake of others. I would be angry, trying to save face for their benefit. It was also out of fear because I knew the only reaction I was going to get was frustration, it wouldn’t be compassion, and I would just feel worse about it all.”

Feelings of burdensomeness and guilt were barriers to many participants reaching out for support. To avoid distressing others, many participants decided not to seek help.

“Even through the darkest times, when I thought I was going to reach out, thoughts of being a burden to my friends would stop me. I couldn’t burden them with that.”

“I’ll be in the middle of a mental breakdown and really upset, angry, crying … but if I sense someone around me, I cover it up. I guess because I am more of the type of person who likes to help people, rather than other people helping me because then I feel like guilty in some weird way. So if anyone would approach me, my entire demeanor would change … I don’t know. I got really good at not looking like I had just been crying two seconds ago, so they would just kind of believe me. It’s like I wanted someone to know, until the actual possibility of someone knowing arose. It’s like, ‘oh man people should really know about this!’ But then as soon as someone asks about it, you’re like, “wait I don’t actually want to tell them.” It sounds like a good idea in theory.”

Some participants expressed concern regarding the possibility of being hospitalized if they disclosed their suicidal ideation. Several individuals had prior experience being on an inpatient unit and/or knew someone who was admitted and had an unhelpful experience.

“The hospital, like it just felt like they just wanted to put me on medication and not give me the resources I felt like I needed which was, I don’t know … maybe dealing with different things like maybe groups or something. This hospital that I went to particularly didn’t really have that. It just had like one substance abuse and mental illness group … and I was like well I don’t struggle with drinking or alcohol, but I went to every group. It just wasn’t helpful and I basically just sat around and colored or was in my room … it just wasn’t helpful.”

Some participants ultimately decided they wanted help; however, many weren’t sure to whom they should turn. As a result, time and effort was devoted to convincing others that they didn’t need support. They explained that this denial was to avoid invalidation, making things worse, and/or feeling like a burden.

“It was really hard for people to wrap their heads around me having lost it so much, and become severely mentally ill. They just couldn’t believe it. There were times when I would really try to pull things together and act like I was ok, and I could get away, you know, I could do that. I could do that for a few weeks, and then you know something would happen.”

“So for me, that person that says they’re okay when a lot of **** is going on in their life right now, those are the ones you want to keep your eye on. The ones that are like, ‘Oh, everything is fine!’ because one night they are going to be alone and feel really, really low and thinking about everything that is going wrong. If they told everyone they are doing okay, they don’t have anyone to call because they just told everyone in their life they are fine. They told everyone.”

Others ultimately decided that they didn’t want help and wanted to die. As a result, participants tried to suppress any signs that indicated their intent to ensure that no one would try to change their mind.

“But for me, I wasn’t going to give any warning signs; I wasn’t going to give anyone the chance to stop me … like if someone really has the intent of killing themselves, they’re not going to give their stuff away, they’re not going to give off any red flags, they’re going to act like everything is normal and they’re going to go home and they’re going to do it. People who are willing to take that step and kill themselves are the best actors in the world.”

Once participants realized they would no longer be in pain, several reported a sense of calmness, resulting in participants being less likely to reach out for help.

“I felt to myself … a sense of peace … of knowing like I am not going to have to worry about this next weekend. I was so peaceful; I woke up in the morning and was like, ‘wow this is amazing.’ I was looking at the sun and the trees and thinking to myself, ‘this is the last time I am going to see this stuff,’ but I was okay with it. I gotta tell you, it’s like you have so much pressure on you and then someone releases the valve because when you finally decide you’re going to do it, it’s like relieving all of the pressure, the pain, the hurt, the everything. It all just goes away.”

#### Lack of intervention leading to disappointment

Regardless of whether participants wanted their attempt to be predictable, seven participants experienced a sense of disappointment when they believed no one in their life intervened prior to their attempt. In some cases, this disappointment intensified the level of distress and may have contributed to the attempt itself.

“Nobody asked me, ‘Hey are you suicidal?’ It didn’t happen but maybe I would have been like, okay, well maybe I should go to the hospital or maybe I can’t do it by myself … But looking back, if somebody did do that, or said like, I mean like even if they said to me, ‘Hey is something wrong?’ I probably would have said no. But, maybe if they did say like, ‘hey are you okay? Are you thinking about hurting yourself?’ Or ‘maybe you should talk to somebody,’ I might have thought about it.”

Although many individuals reported available social networks, this wasn’t enough; participants yearned for individuals in their environment to do more.

“They would say, ‘If you ever need me I’m always here.’ But then when you call them and they don’t answer. And you’ve called everybody in your phone book, and nobody answers. You start feeling like, ‘Well, I don’t want to be a nuisance to people.’ There are people who were failed by so many people in their lives. There were signs … many, many signs … and we need to teach people how to see them and get involved.”

“I wanted help. I wanted to get help. And that’s why I was telling them, I mean I had a plan and I wanted to do it, but I wanted help the same way. I wish they would have told an adult or called like a lifeline. You know something like that.”

#### Difficulty communicating

Six participants expressed difficulty in directly communicating about their suicidal ideation to others. Thus, even if they did want help, participants were unsure how to effectively obtain it.

“My teachers didn’t really understand me and it was hard to communicate with them. They knew something was wrong but they couldn’t pinpoint it. I believe I wanted help, I just didn’t know how to communicate it … they just wanted me off the grounds. I would get upset and get detention. I would purposely do things and then get suspended and was like, ‘oh, yay, I have vacation.’ It was messed up but I didn’t know how else to tell them.”

At times, many participants struggled to identify what they were feeling which made it even more challenging to communicate with others.

“I probably wasn’t a hundred percent honest, not like trying to be deceptive but sometimes I didn’t even recognize the things I was feeling. I didn’t want to have to explain it to anyone. I was like, ‘I don’t understand this, I don’t want to try to have to explain it to someone else.’”

Although participants found it challenging to describe their experience, many reported directly speaking with others about their intent to die. However, this type of communication was perceived as being ineffective as it was frequently met with lack of compassion.

“There would be an intense sense of frustration. Much like you would expect a stereotypical fictional mother to react to her daughter impulsively cutting off most of her hair and dying the rest of it bright orange … “Why would you go and do that?! It’s so stupid!” Stuff like that; same tone and treatment of the situation. They thought it was more just a cry for attention.”

At times, this form of direct communication about suicidal urges was also met with seemingly unintentional invalidation. Although participants often acknowledged that no malicious intent may have been present, invalidating responses were still detrimental to them.

“I’d be like, ‘I just can’t handle this. I’m so depressed and I just, I don’t know how to deal with this anymore … and I don’t want to live like this.’ And they’d be like, ‘Oh, you’re just having a day. Why don’t we go get a pedicure?’ I know they didn’t mean any ill will towards me, but I think they genuinely thought I was stronger than I was, and I think they genuinely just thought like, ‘She’s just having a bad day. You know tomorrow she is going to be all rainbows and sunshine, and talk it out … ’ and I really wasn’t there. I was really like, I don’t think I can handle this anymore.”

## Discussion

To date, suicide prevention initiatives have focused on spreading awareness of warning signs, risk factors, and action plans to the general public (AAS, 2017; Arensman, 2017; Dedić, 2016; Rudd et al., 2006). Although previous research has shown this approach to be effective in enabling individuals to more easily identify individuals at risk (Lester et al., 2011) suicide rates are still on the rise (CDC, 2018). Thus, a different approach may be needed. This exploratory qualitative study sought to gain a better understanding of the complexity of predicting suicide by speaking directly to individuals with lived experience. Consistent with previous literature highlighting the cost-benefit determination suicidal individuals consider before disclosing their ideation or intent (e.g., Frey, Fulginiti, Lezine, & Cerel, 2018; Sheehan et al., 2019), our results suggest there is a notable disconnect between attempters and their environment.

The superordinate theme of a disconnect between attempters and their environment was first indicated through a sense of ambivalence regarding whether or not participants wanted help prior to their attempts, which is consistent with prior research (Bruffaerts et al., 2011; Czyz et al., 2013). This ambivalence was attributed to fears of being stigmatized, feelings of burdensomeness, feelings of invalidation, and not knowing whom to turn to while in a crisis. As a result, participants frequently suppressed the exhibition of warning signs. This theme supports both the biosocial theory and the interpersonal theory of suicide. In line with the biosocial theory (Linehan, 1993), many participants reported having family members with histories of mental illnesses (i.e., biological vulnerability) and perceived their environments to be invalidating (i.e., dysfunctional or invalidating environment). The consequences of this invalidating environment suggest possible reasons for the difficulty of predicting suicide deaths and the national increase in suicide fatalities. If an attempter perceives their environment to be invalidating, our results suggest this may lead to an increased urge to conceal warning signs and even attempt suicide to avoid feeling worse. This is consistent with previous studies of suicide attempt survivors that have found fears about receiving unsupportive responses, being rejected, and being stigmatized as primary reasons preventing disclosure (e.g., Frey et al., 2018; Sheehan et al., 2019). Some individuals might also interpret their environment to be invalidating after the exhibition of warning signs is not taken seriously. These indications are consistent with Latakienė et al. (2016) findings that 1) most suicide attempters were only taken seriously during their attempts, but not before and after an attempt and 2) non-responsive reactions resulted in heightened urges to die by suicide.

The importance of the interpersonal theory of suicide (Joiner, 2005) in understanding the current dilemma was partially supported. Several participants mentioned feelings of burdensomeness as a barrier to expressing signs of imminent danger to individuals in their lives. Thus, many individuals chose to mask their suffering by obscuring warning signs and did not reach out for support. Participants also reported a sudden change after their first attempt that was described in terms of a “line being crossed” which could be understood as evidence for acquired capability. This theme is consistent with recent research showing that feeling like a burden is a common response to friends or family members’ reactions to disclosure by attempt survivors (e.g., Fulginiti & Frey, 2018). Taken together, prior research and our results suggest that suicidal individuals’ concerns about feeling burdensome if they disclose their suicidal thoughts or intent may be well-founded. Contrary to the theory, study participants did not report thwarted belongingness.

The reported disconnect between attempters and their environment was also indicated through disappointment following a lack of intervention. Although the majority of participants struggled with the decision to seek help prior to an attempt, most participants still hoped that someone would prevent their attempt. This suggests that the “actors” may have attributed their attempt to the lack of intervention from “observers” around them, which they may perceive as a situational factor. Although this study only received the accounts of the “actors,” participants perceived that the “observers” in their environments appeared to rarely recognize warning signs or communication about suicidal ideation as serious. At times, individuals in the environment interpreted signs as “attention-seeking,” much like an observer tending to attribute causes (i.e., an individual exhibiting warning signs) to personal characteristics. As a result, participants reported that they ultimately began to suppress warning signs and lose hope in others preventing their attempt. These results are consistent with Veiel et al. (1988) findings that an available social network is not enough for participants to feel validated and supported; instead, an appropriate crisis response from the social network was needed. Wolk‐Wasserman’s (1986) also highlighted the deleterious effects of significant others responding to communication of risk with silence, rather than intervention.

The demand for the environment to aid suicidal individuals and, at the same time, individuals masking their need for support also provides evidence for the dialectical dilemma encountered by providers working with suicidal patients of “active passivity versus apparent competence” (Linehan, 1993). This dilemma suggests that in certain situations, suicidal individuals indicate to their environment that they have the ability to handle stressors (e.g., an attempter suppressing warning signs and seeming to function normally), yet in different situations they act helpless (e.g., an attempter yearning for individuals in their environment to prevent their attempt). This results in a misunderstanding between attempters and individuals around them, which might contribute to the difficulty of predicting suicide and result in ambiguous warning signs (Robins et al., 1959). Although individuals in attempters’ environments might not be given enough information to realize intervention is warranted and attempters might be suppressing warning signs to avoid worsening their situation, the lack of intervention is enough to exacerbate the urge to die.

The reported disconnect between attempters and their environment was also indicated through difficulty directly communicating to others about thoughts of suicide. Many participants found it challenging to identify what emotions they were experiencing and thus, did not know how to describe them to others. While this particular barrier to disclosure or exhibition of warning signs has received minimal attention, it does fit within theoretical conceptualizations of suicidal behaviors as resulting from deficits in emotional awareness and regulation (e.g., Linehan, 1993). Additionally, although some participants directly spoke about their suicidal ideation, reactions were predominantly unfavorable. Others invalidated participants’ experiences either by not taking them seriously and/or by trying to be supportive and subsequently invalidating participants (e.g., by remaining optimistic and minimizing their suffering). This description of invalidation is consistent with previous research suggesting that frequent reactions to suicidal communication are disbelief, denial, avoidance, or attempts to interact as if nothing has happened (Cowgell, 1977; Rudestam, 1971) and that the reactions of friends and family members (e.g., stigmatizing statements, avoidance) can exacerbate negative experiences in the suicide attempter (Frey, Hans, & Cerel, 2017). As a result, many participants decided to suppress warning signs to cope with their suffering alone and not have to worry about it getting worse because of others’ unpredictable reactions. Participants also used less direct approaches to communicating about suicidal ideation to gauge others’ reactions (e.g., “I can’t do this anymore”). However, this approach often inhibits an accurate interpretation of the message, leads to misunderstanding, and consequently prevents the proper response and suicide prevention intervention (Owen et al., 2012).

In summary, these themes underscore the complexity of predicting and preventing suicide, highlighting the role that complex decisions about communication by the suicidal individual may play in difficulty predicting suicide attempts and deaths. Crucially, engaging individuals with lived experience in the process provides an opportunity for greater understanding and more effective outreach and intervention approaches. Our results highlight that one complication of predicting suicide is individual tendencies to suppress and/or obscure warning signs. As identified in previous studies with suicide attempt survivors, fear of stigmatization and feelings of burdensomeness and guilt emerged as important themes for our participants. Additionally, difficulty communicating with others and lack of crisis support were identified as barriers to help-seeking behaviors.

Several limitations of the current study should be taken into consideration when interpreting the findings. First, the results may be unique to the sample of the present study (e.g., 93% female, 90% Caucasian, willing to speak about experiences prior to their first suicide attempt), reducing generalizability. In particular, the characteristics of suicidal behaviors in men and women and in White and non-White populations are substantially different (e.g., Bhui, Dinos, & McKenzie, 2012; De Luca, Yan, Lytle, & Brownson, 2014). Particularly in relation to rates of disclosure of suicidal ideation or intention and exhibition of warning signs, females are more likely to seek help for suicidal thoughts and behaviors (e.g., Czyz et al., 2013). Although the limited diversity in our sample precludes broad generalizations beyond the study sample, rates of suicide in the US have risen across gender and ethnic groups (CDC, 2018) and the male and non-White participants’ responses were consistent with the themes throughout the full sample, lending support to their inclusion in the present analysis. Additionally, although 7 out of 15 participants reported high-lethality attempts, the fact that all participants attempted suicide (and did not die as a result of the attempt), may limit the generalizability of our findings to suicidal individuals who had different experiences exhibiting warning signs that ultimately prevented their suicide attempts or who made less lethal suicide attempts. Second, participants were required to rely on their memory when discussing experiences prior to their first suicide attempt, which might have occurred several years ago, resulting in possible response and recollection biases. To address this limitation, participants were asked to indicate the clarity of their memory for relevant experiences. All participants reported at least a moderate (3 out of 5) level of clarity. However, a previous study found that suicide attempters who presented to emergency rooms in Korea (Lim et al., 2014) were more likely to attribute their attempt to environmental factors than psychopathology; however, interviewers attributed the attempts more to psychopathology than the environment. This may suggest that relying exclusively on retrospective reports from suicide attempters may be biased by the attempter’s perception or memory; however, other studies have suggested that recall may not be impaired or more biased in previously suicidal samples. For instance, Chu, Buchman-Schmitt, and Joiner (2015) found that suicide attempters recalled events using more first-person, internally-focused perspectives (i.e., more detailed) than controls without a suicide attempt history. Thus, future research will be needed to determine the extent to which biased recall might account for our findings.

A third limitation is that participants were excluded if they had suicidal thoughts in the previous month to mitigate potential risks of participation; however, this exclusion criterion might have further limited the scope of available information. Fourth, although IPA is a valid form of data analysis, it requires subjective interpretations of participants’ experiences, leading to possible misunderstandings and/or biased appraisals.

In the future, research should continue to focus on reducing the disconnection between attempters and their environments. In addition to educating the public about risk factors and warning signs of suicide, identifying skills to facilitate communication about suicidal thoughts and/or urges between suicidal individuals and their support networks is crucial. It is also imperative that future research focuses on treatment development targeting burdensomeness and invalidation. As a result, suicidal individuals might feel less ambivalent about seeking help and may no longer feel the need to suppress and/or obscure warning signs, ultimately increasing the predictability of suicide.
